# Supplementary material for: Sequential Deactivation Across the Hippocampus‐Thalamus‐mPFC Pathway During Loss of Consciousness
Source: Adv Sci (Weinh). 2024 Sep 9;11(42):2406320. doi: 10.1002/advs.202406320 (PMC11558098; doi:10.1002/advs.202406320)
Supplement: Supplementary file 1 — Supporting Information [file ADVS-11-2406320-s001.pdf]

ADVANCED SCIENCE
Open Access

Supporting Information

for *Adv. Sci.*, DOI 10.1002/advs.202406320

Sequential Deactivation Across the Hippocampus-Thalamus-mPFC Pathway During Loss of Consciousness

*Xiaoai Chen, Samuel R. Cramer, Dennis C.Y. Chan, Xu Han and Nanyin Zhang**

Supplementary Figures

Title: Sequential deactivation across the hippocampus-thalamus-mPFC pathway during loss of consciousness

Authors: Xiaoai Chen[a], Samuel R. Cramer[b], Dennis C.Y. Chan[a], Xu Han[a], Nanyin Zhang[a,b,c,d*]

Affiliations:

a. Department of Biomedical Engineering, The Pennsylvania State University, University Park, USA
b. The Neuroscience Graduate Program, The Huck Institutes of the Life Sciences, The Pennsylvania State University, University Park, USA
c. Center for Neurotechnology in Mental Health Research, The Pennsylvania State University, University Park 16802, USA
d. Center for Neural Engineering, The Pennsylvania State University, University Park 16802, USA

***Corresponding author:** Nanyin Zhang, Ph.D. – nuz2@psu.edu

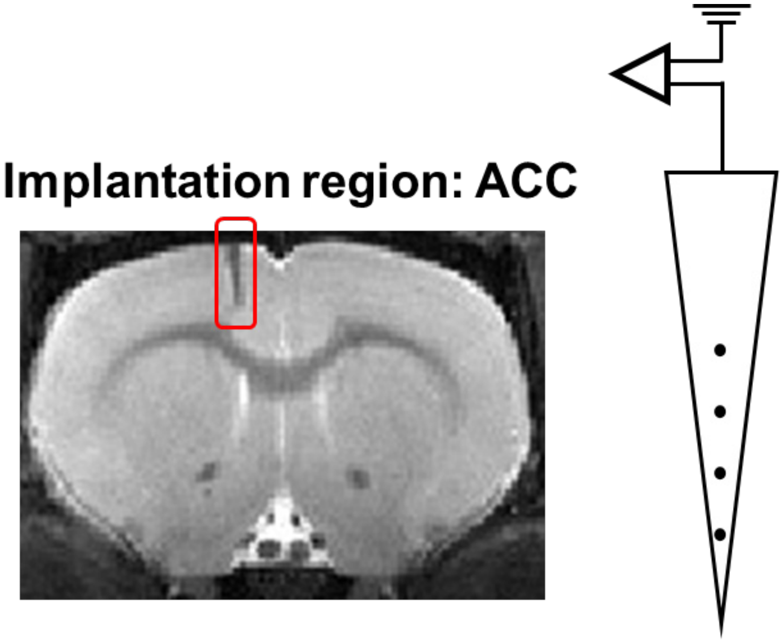

Figure S1. An exemplar T2-weighted image showing an MR-compatible electrode implanted at rat's left ACC.

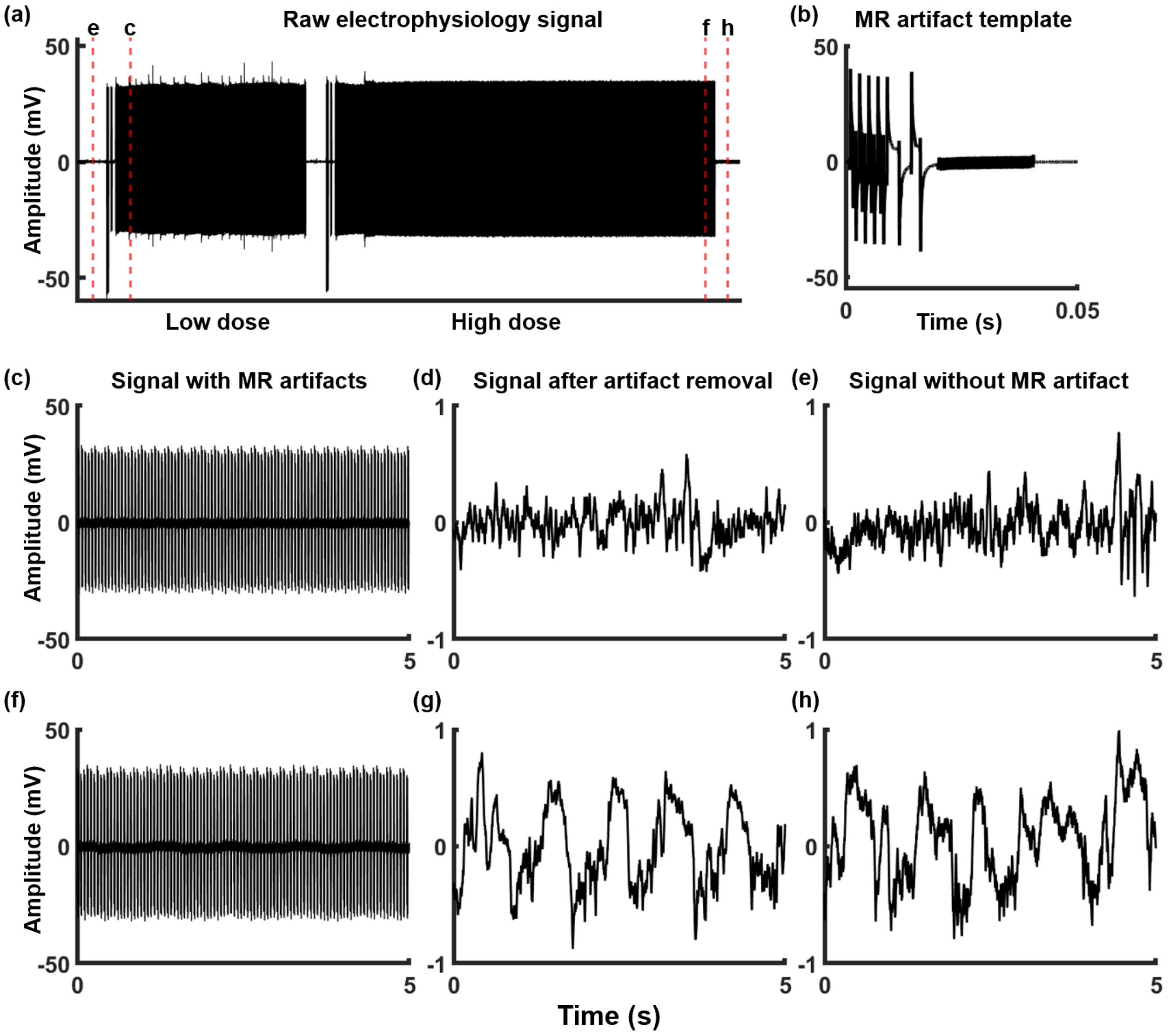

Figure S2. **Removing MR-induced artifacts from simultaneously recorded electrophysiology signal.** (**a**) Raw electrophysiology signal of an exemplar scan. The electrophysiology signal was continuously recorded from approximately 2 min before low-dose fMRI scan to 2 min after high-dose fMRI scan. (**b**) A noise template of MR-induced artifacts from a 20-s time window. (**c,f**) Raw electrophysiology signal with MR artifacts in the 5-s windows under low dose and high dose of propofol, as marked by red dashed lines in (a). (**d,g**) LFP after denoising MR artifacts in the same 5-s windows as (**c,f**). (**e,h**) LFP without MR artifact in the 5 s windows under low dose and high dose of propofol, as marked by the red dashed lines in (**a**).

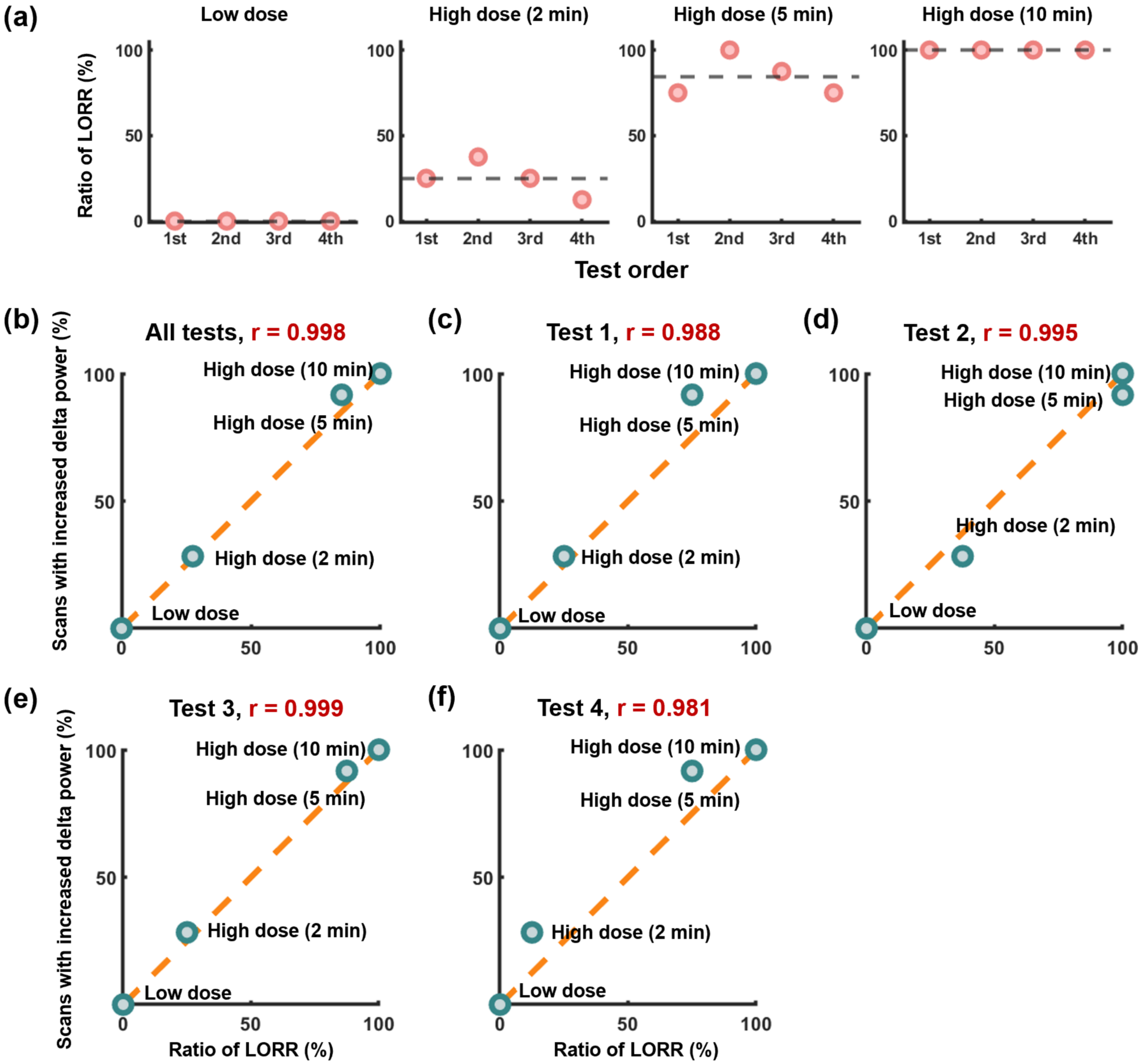

Figure S3. Assessment of the reproducibility of LORR. (**a**) LORR tests were repeated four times in a separate group of animals (n = 8). The ratio of animals exhibiting LORR under different dosing conditions was calculated for all four tests. Dash lines indicate the ratio obtained when lumping data across all 4 tests for each condition. (**b-f**) Correlation between the percentage of animals exhibiting LORR at various time points following high-dose infusion (**b**) across all tests, and (**c-f**) in tests 1–4 and the percentage of scans showing the onset of low-frequency LFP power increase within the corresponding time intervals.

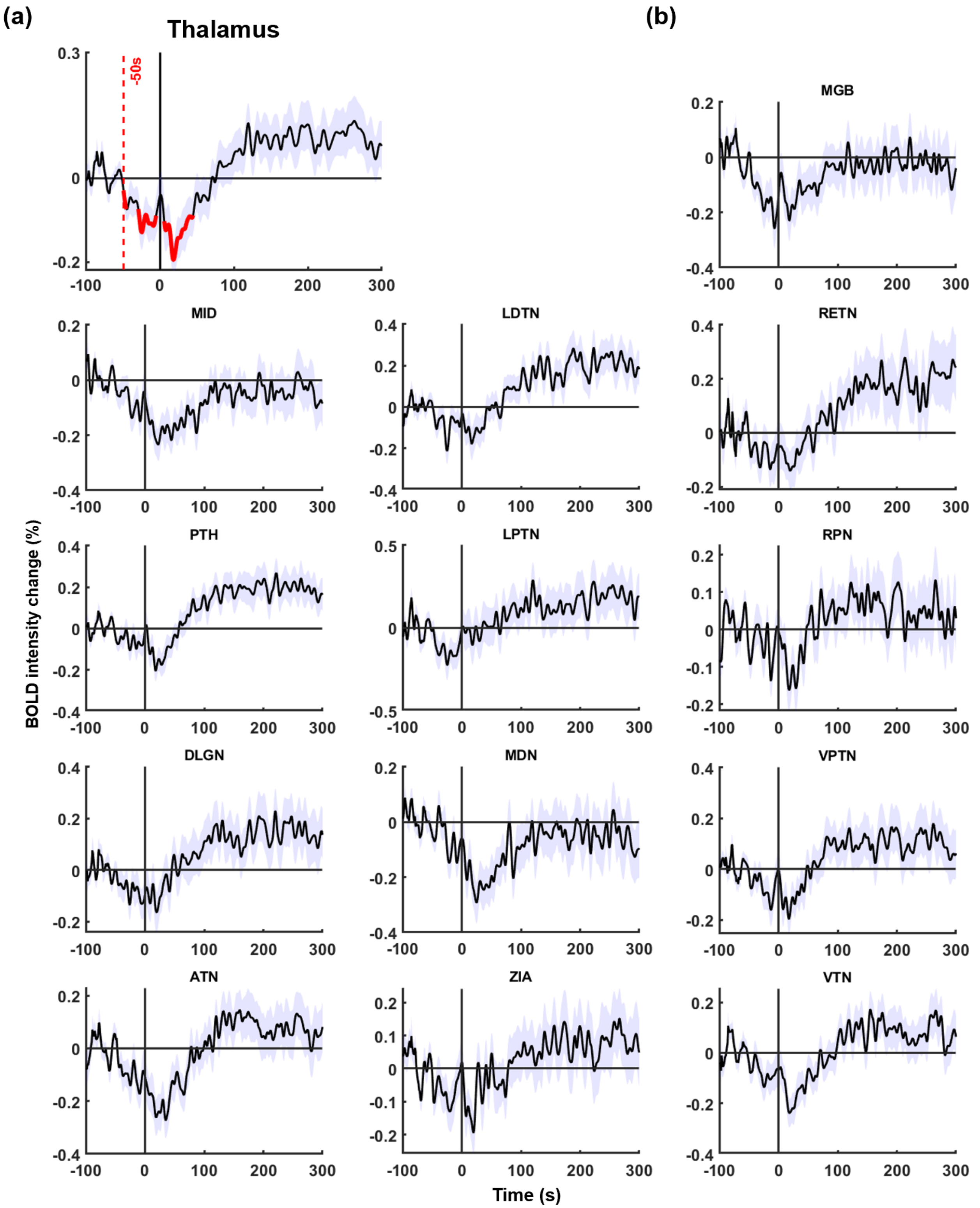

Figure S4. BOLD intensity changes around the onset of LOC in subregions of the thalamus. (**a**) BOLD intensity change in the thalamus (the same as Fig. 3b). (**b**) BOLD intensity changes of individual nuclei within the thalamus. From left to right, top to bottom: medial geniculate body

(MGB), midline thalamic nucleus (MID), laterodorsal thalamic nucleus (LDTN), reuniens thalamic nucleus (RETN), posterior thalamic nucleus (PTH), lateral posterior thalamic nucleus (LPTN), reticular (pre)thalamic nucleus (RPN), dorsal lateral geniculate nucleus (DLGN), mediodorsal thalamic nucleus (MDN), ventral posterior thalamic nucleus (VPTN), anterior thalamic nucleus (ATN), zona incerta (ZIA), and ventral thalamic nucleus (VTN).

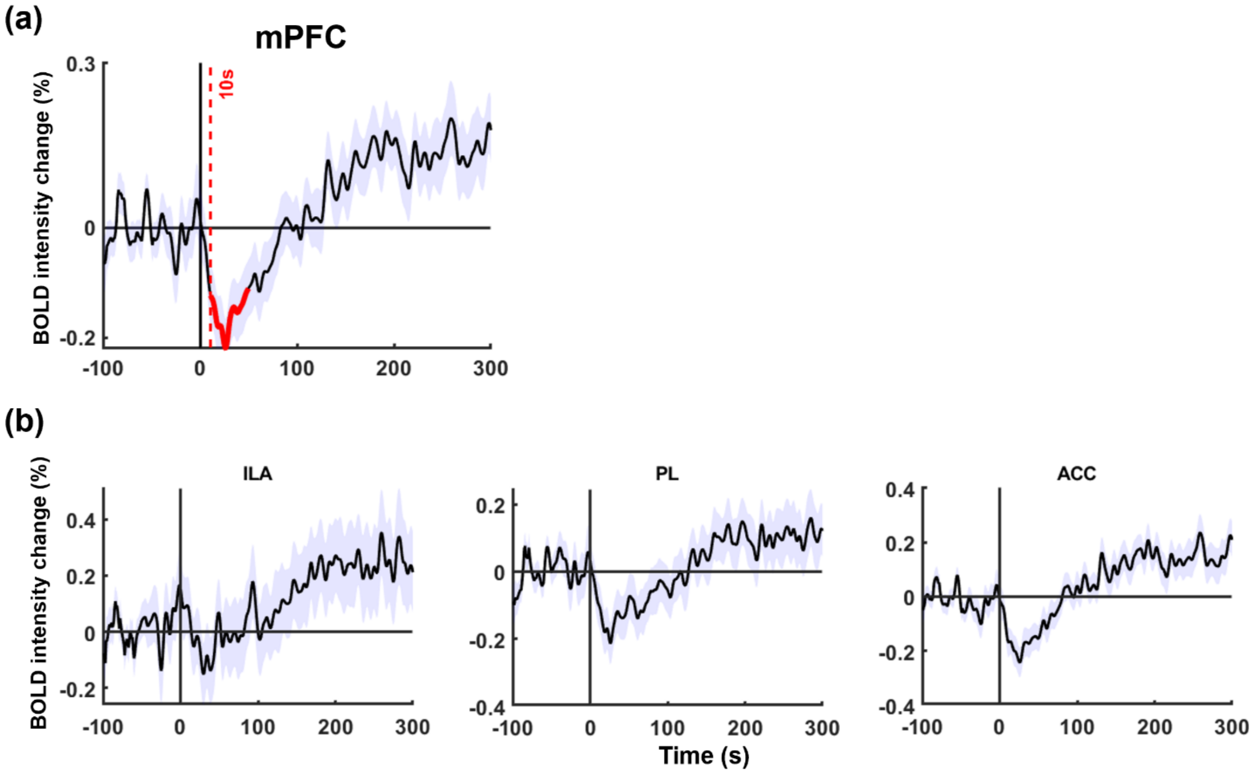

Figure S5. BOLD intensity changes around the onset of LOC in subregions of the medial prefrontal cortex. (**a**) BOLD intensity change in the mPFC (same as Fig. 3c). (**b**) BOLD intensity changes of individual subdivisions within the mPFC. From left to right: infralimbic area (ILA), prelimbic area (PL), and anterior cingulate cortex (ACC).

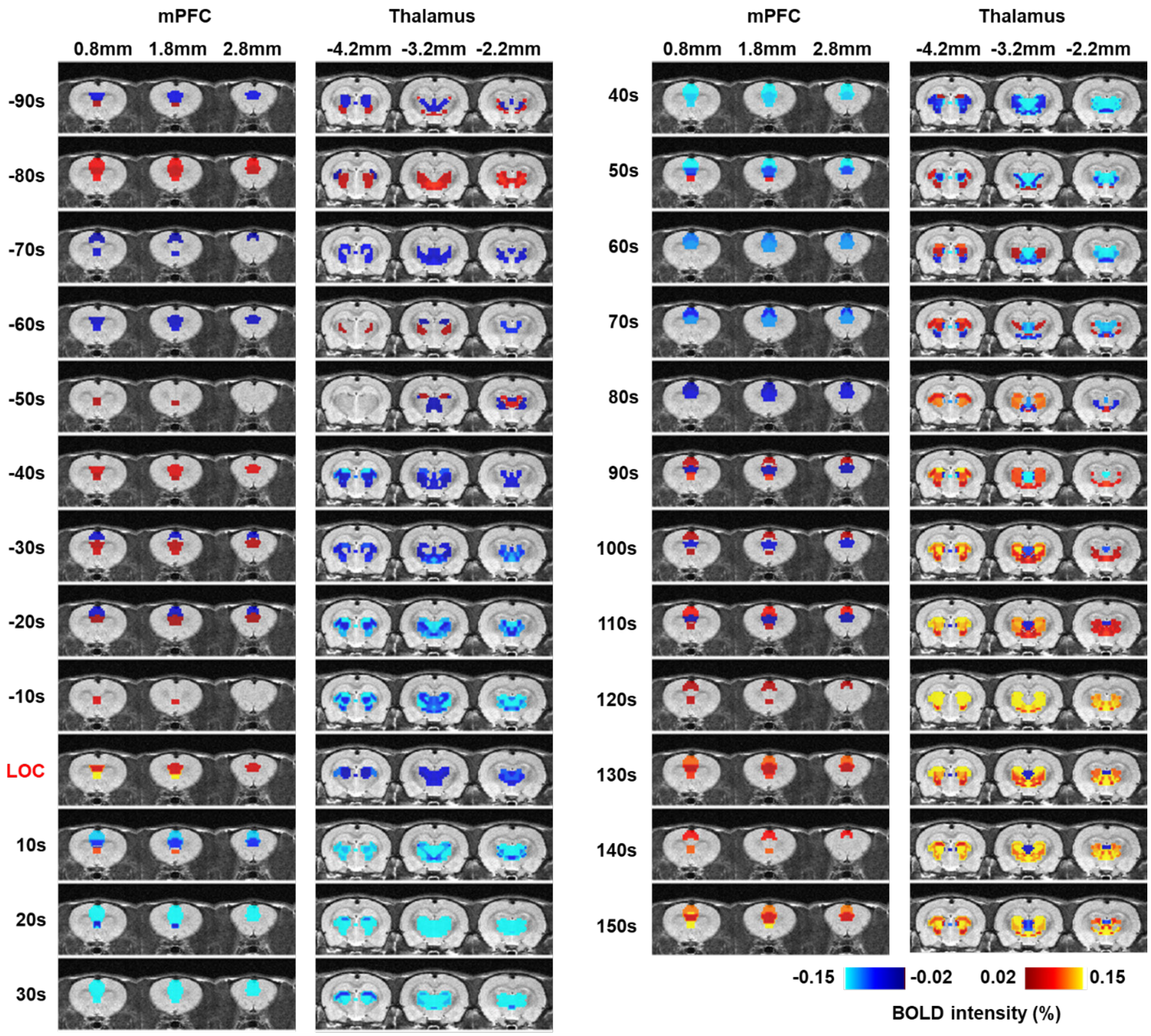

Figure S6. BOLD intensity changes of ROIs in the thalamus and mPFC within the time window of -90 s to 150 s around the onset of LOC. The BOLD intensity is normalized to the baseline level, defined as the averaged BOLD intensity during the period of -100 s to -45 s before LOC.

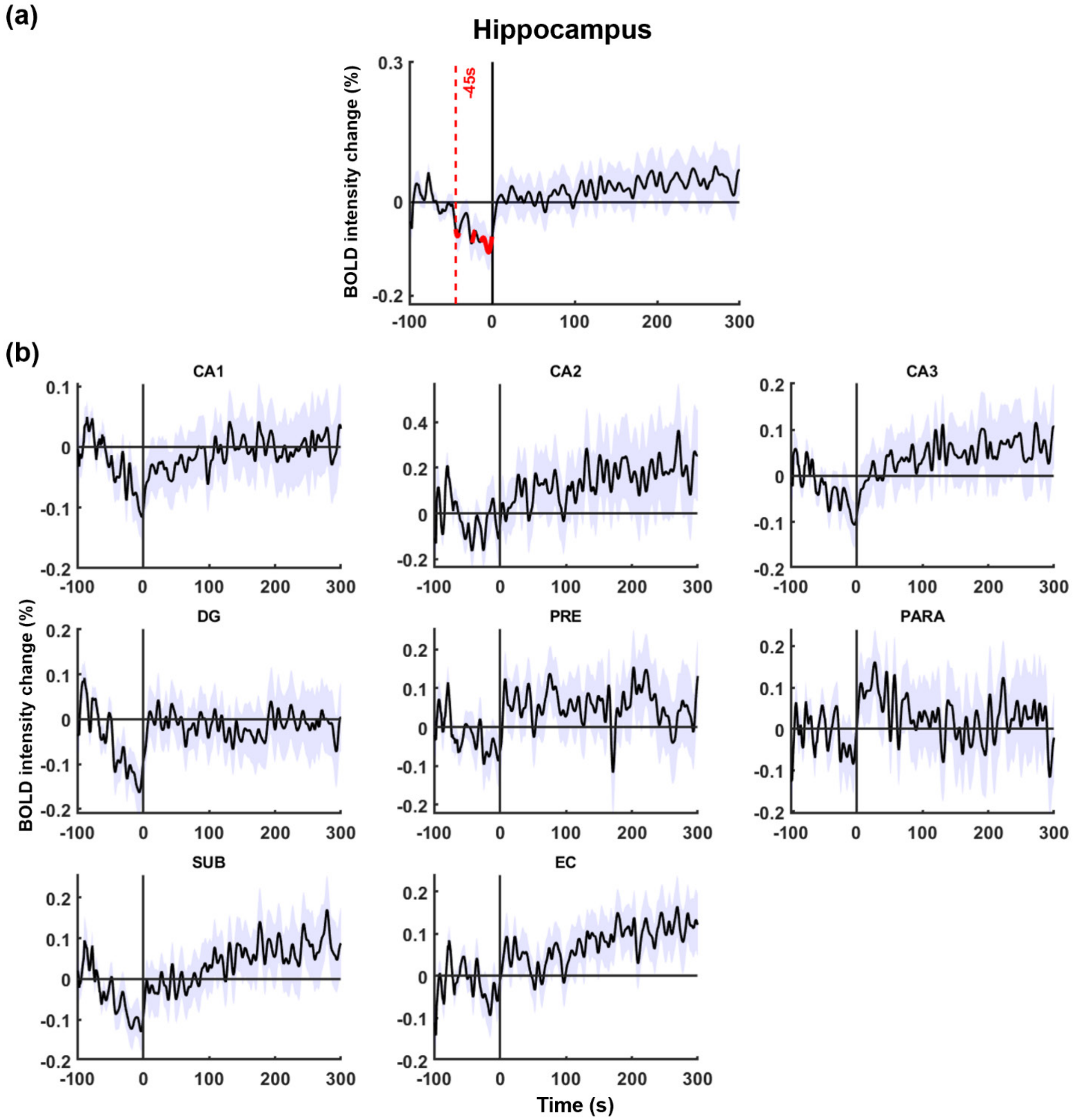

Figure S7. BOLD intensity changes around the onset of LOC in subregions of the hippocampus. (**a**) BOLD intensity change in the hippocampus (the same as Fig. 3f). (**b**) BOLD intensity changes of all subregions within the hippocampus. From left to right, top to bottom: cornu ammonis 1 (CA1), cornu ammonis 2 (CA2), cornu ammonis 3 (CA3), dentate gyrus (DG), presubiculum (PRE), parasubiculum (PARA), subiculum (SUB), and entorhinal cortex (EC).

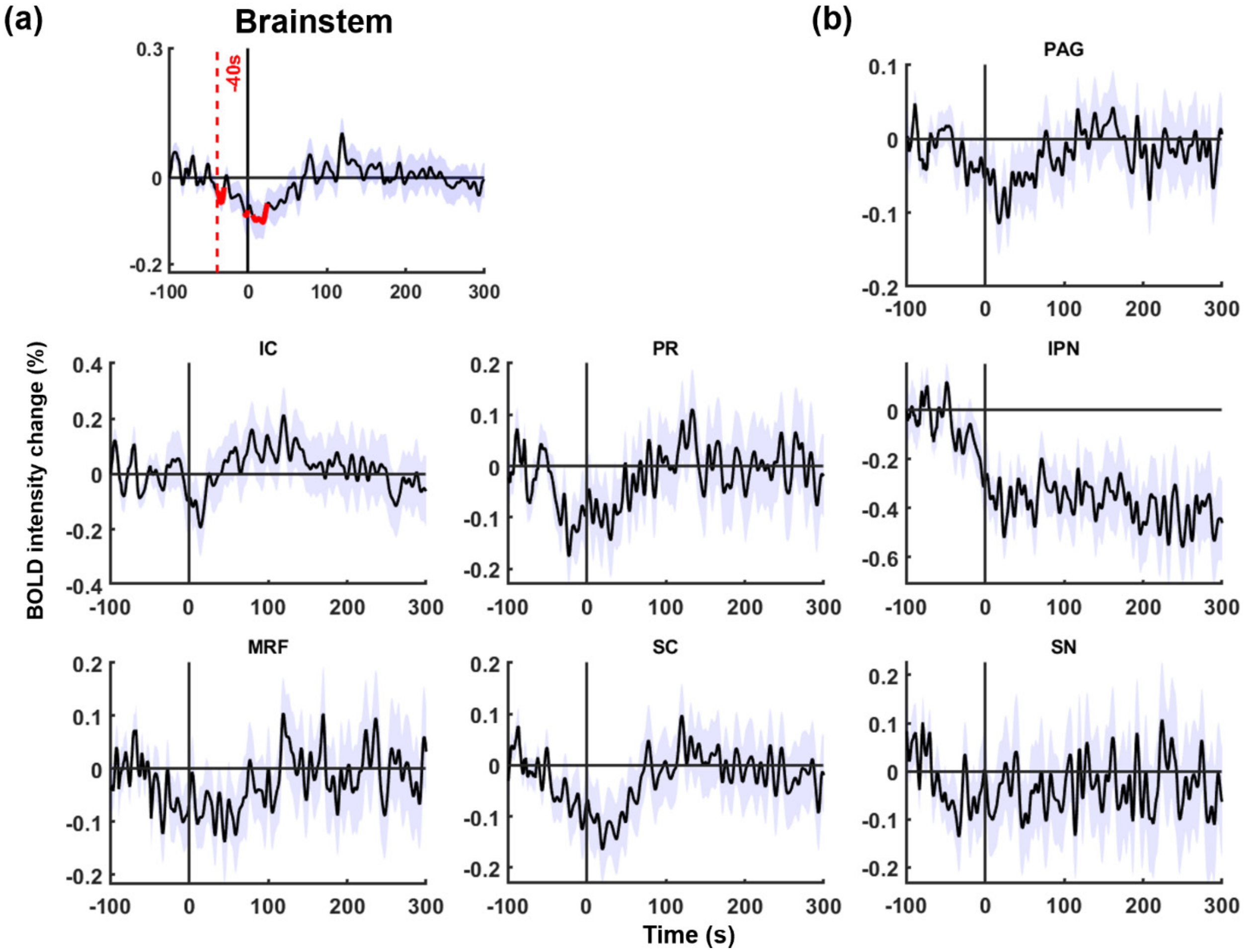

Figure S8. BOLD intensity changes around the onset of LOC in subregions of brainstem. (**a**) BOLD intensity change in the brainstem (the same as Fig. 3g). (**b**) BOLD intensity changes of individual subregions within the brainstem. From left to right, top to bottom: periaqueductal gray (PAG), inferior colliculus (IC), pretectal region (PR), interpeduncular nucleus (IPN), mesencephalic reticular formation (MRF), superior colliculus (SC), and substantia nigra (SN).

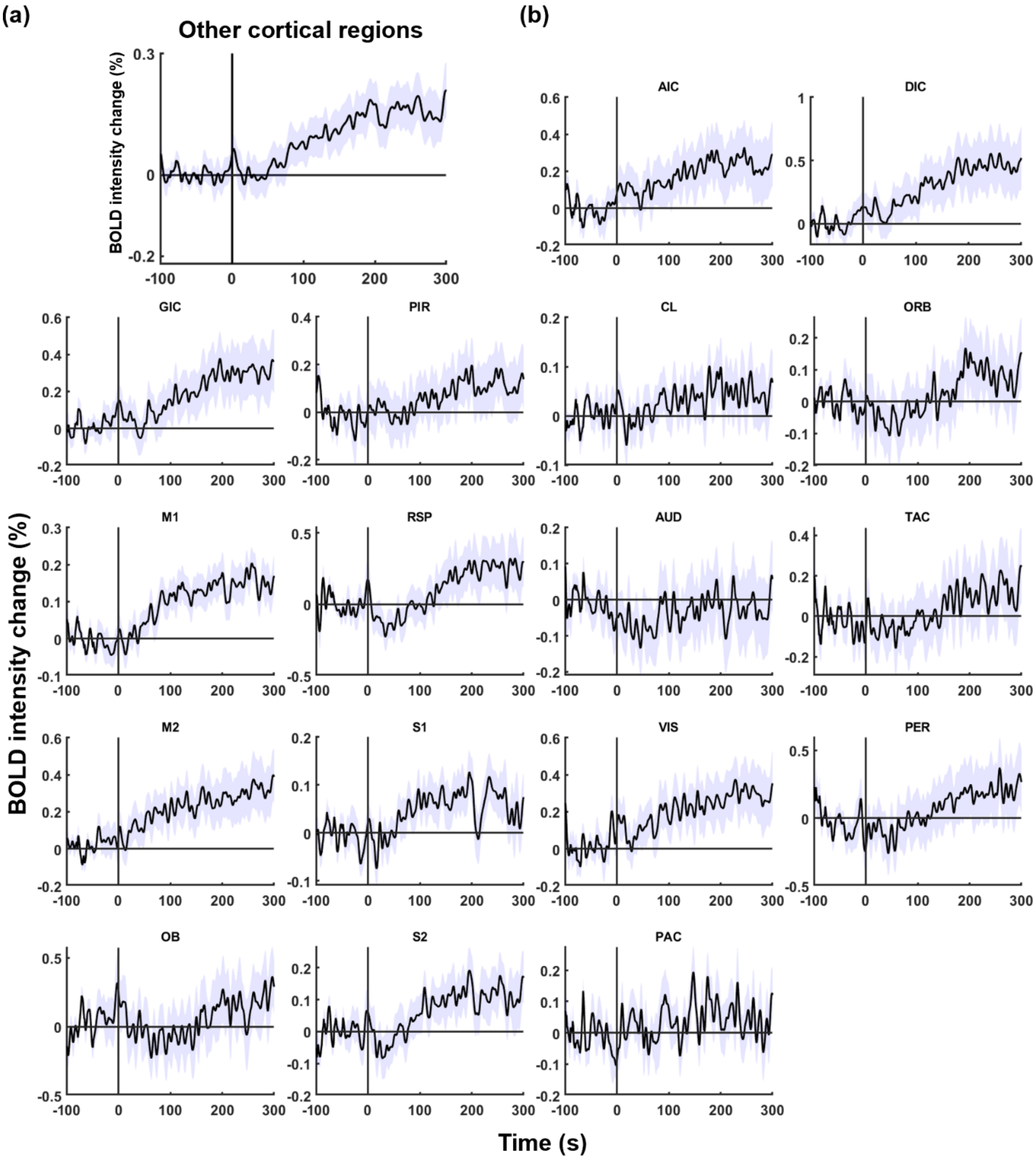

Figure S9. BOLD intensity changes around LOC in other cortical regions. (**a**) BOLD intensity change in cortical regions excluding the mPFC. (**b**) BOLD intensity changes of all ROIs included in the cortex except for the mPFC. From left to right, top to bottom: Agranular insular cortex (AIC), Dysgranular insular cortex (DIC), Granular insular cortex (GIC), Piriform cortex (PIR), Claustrum (CL), Orbital area (ORB), Primary motor area (M1), Retrosplenial area (RSP), Auditory area (AUD), Temporal association cortex (TAC), Secondary motor area (M2), Primary somatosensory area (S1), Visual area (VIS), Perirhinal area (PER), Olfactory bulb (OB), Secondary somatosensory area (S2), Parietal association cortex (PAC).

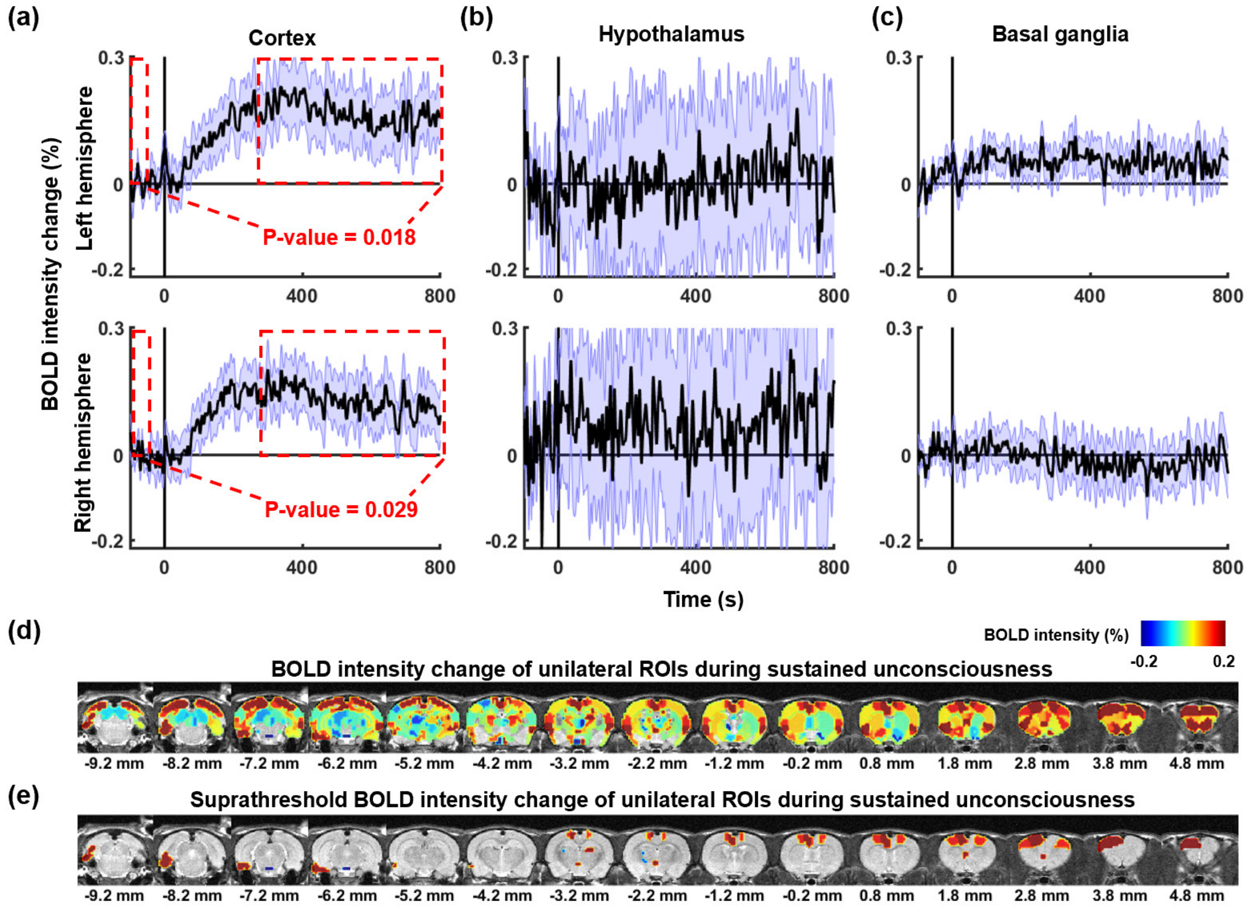

Figure S10. Brain-wide BOLD activity during sustained unconsciousness based on unilateral ROIs. (**a**) The cortex in both left and right hemispheres exhibit a gradual, monotonic increase in the BOLD signal after the onset of LOC, reaching a plateau during sustained unconsciousness (defined as the period 300 s – 800 s post LOC, marked by the red dashed rectangle on the right), significantly exceeding the baseline level (the red dashed rectangle on the left, p value (left hemisphere) = 0.018, p value (right hemisphere) = 0.029). (**b-c**) The b) hypothalamus and c) basal ganglia do not appear to be involved in the process of LOC. (**d**) Map of brain-wide BOLD intensity of unilateral ROIs during sustained unconsciousness. (**e**) Suprathresholded map of (d) ($p < 0.05$).

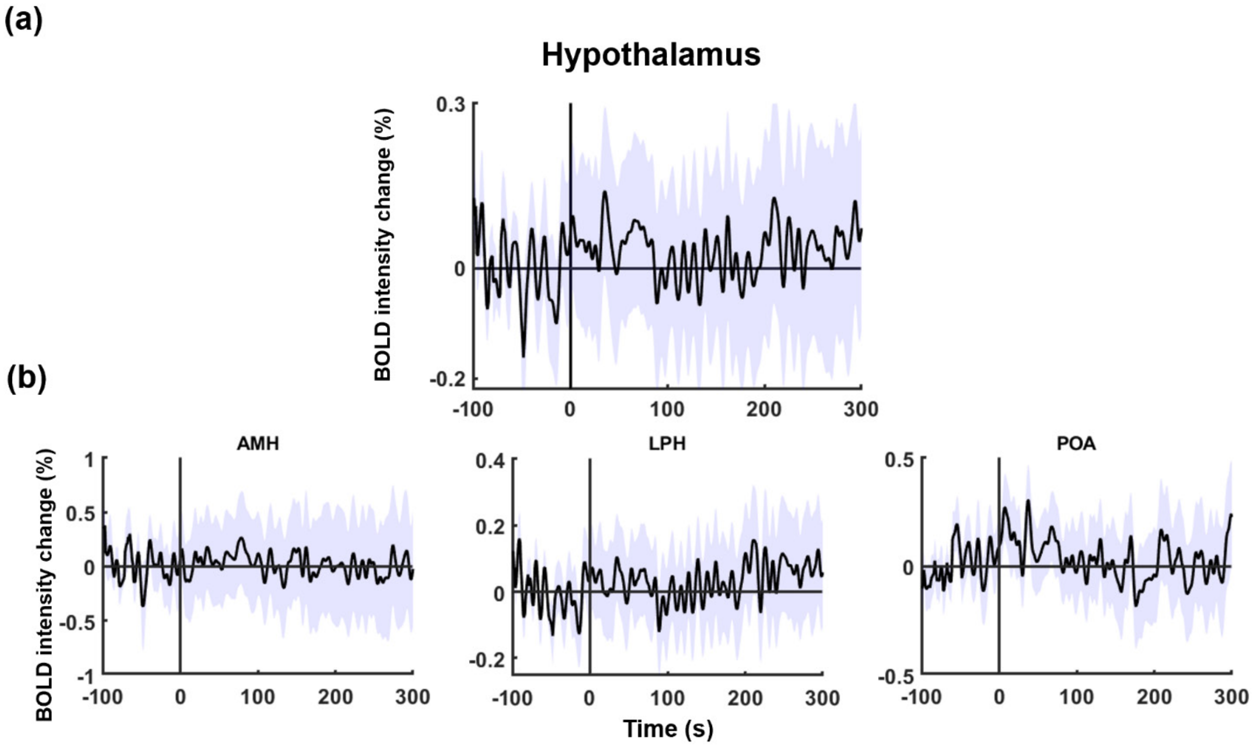

Figure S11. BOLD intensity changes around LOC in subregions of hypothalamus. (**a**) BOLD intensity change in the hypothalamus. (**b**) BOLD intensity changes of all ROIs included in hypothalamus. From left to right: Anterior medial hypothalamus (AMH), Lateral posterior hypothalamus (LPH), Preoptic area (POA).

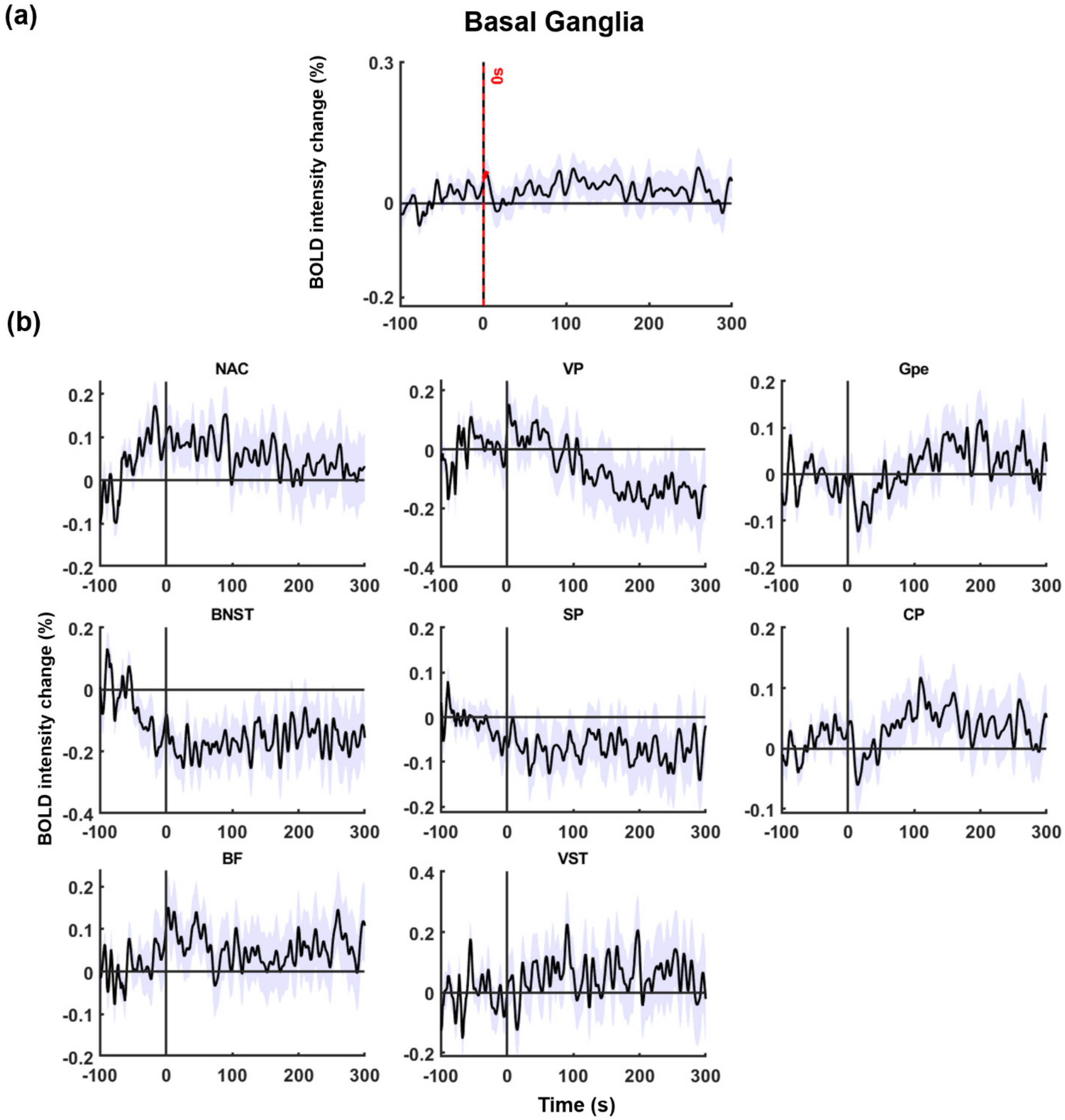

Figure S12. BOLD intensity changes around LOC in subregions of basal ganglia. (**a**) BOLD intensity change in the basal ganglia. (**b**) BOLD intensity changes of all ROIs included in basal ganglia. From left to right, top to bottom: Nucleus accumbens (NAC), Ventral pallidum (VP), Globus pallidus (Gpe), Bed nucleus of the stria terminalis (BNST), Septal region (SP), Caudate putamen (CP), Basal forebrain (BF), Ventral striatal region (VST).

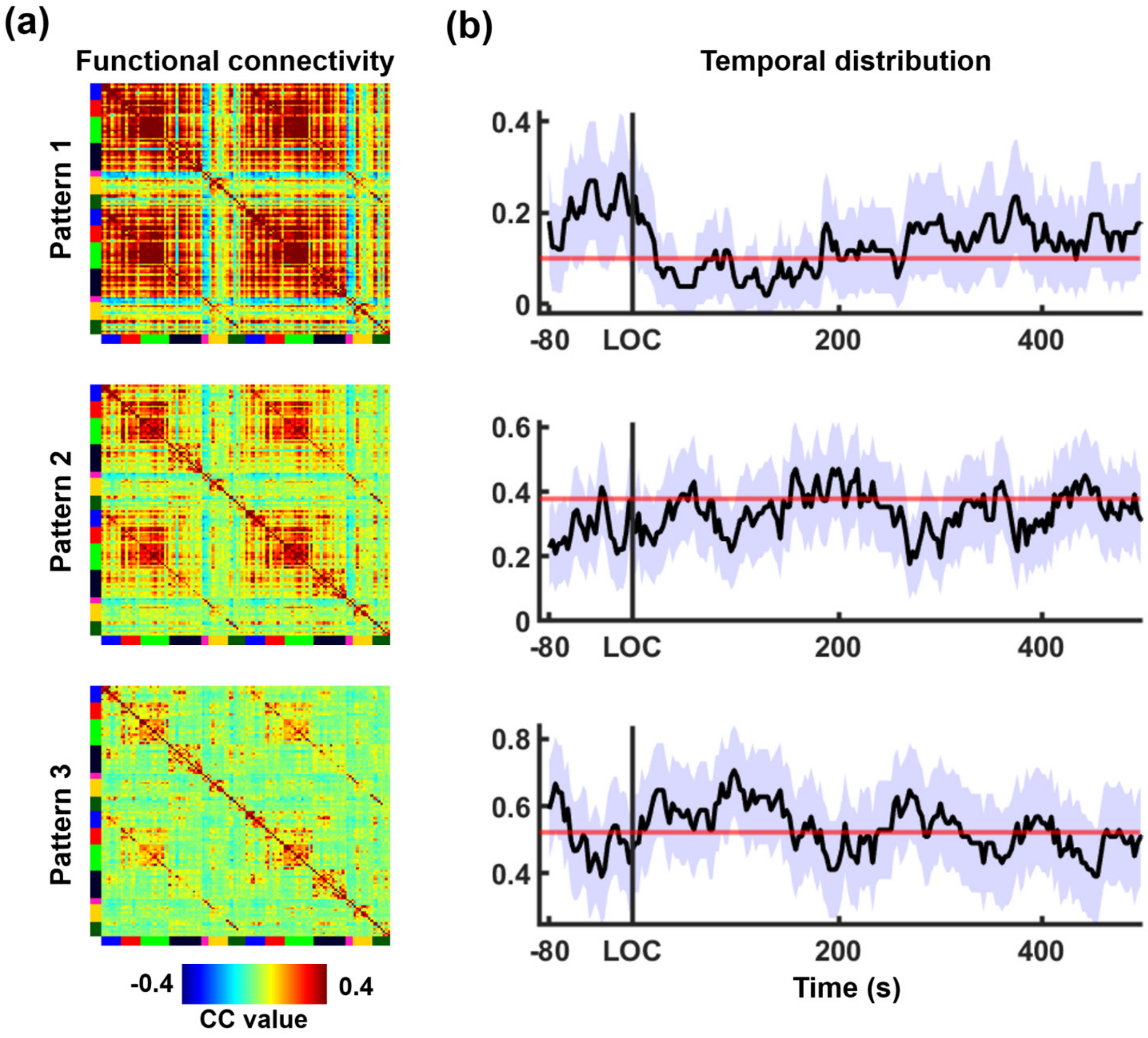

Figure S13. Dynamic brain states under different consciousness conditions with a cluster number of 3. (**a**) Three brain states, identified by unsupervised clustering of dynamic functional connectivity matrices. (**b**) Temporal distributions of three brain states around LOC and under the sustained unconsciousness. The purple shade indicates 95% confidence interval. The red line represents the averaged temporal distribution under low-dose propofol (i.e. baseline).

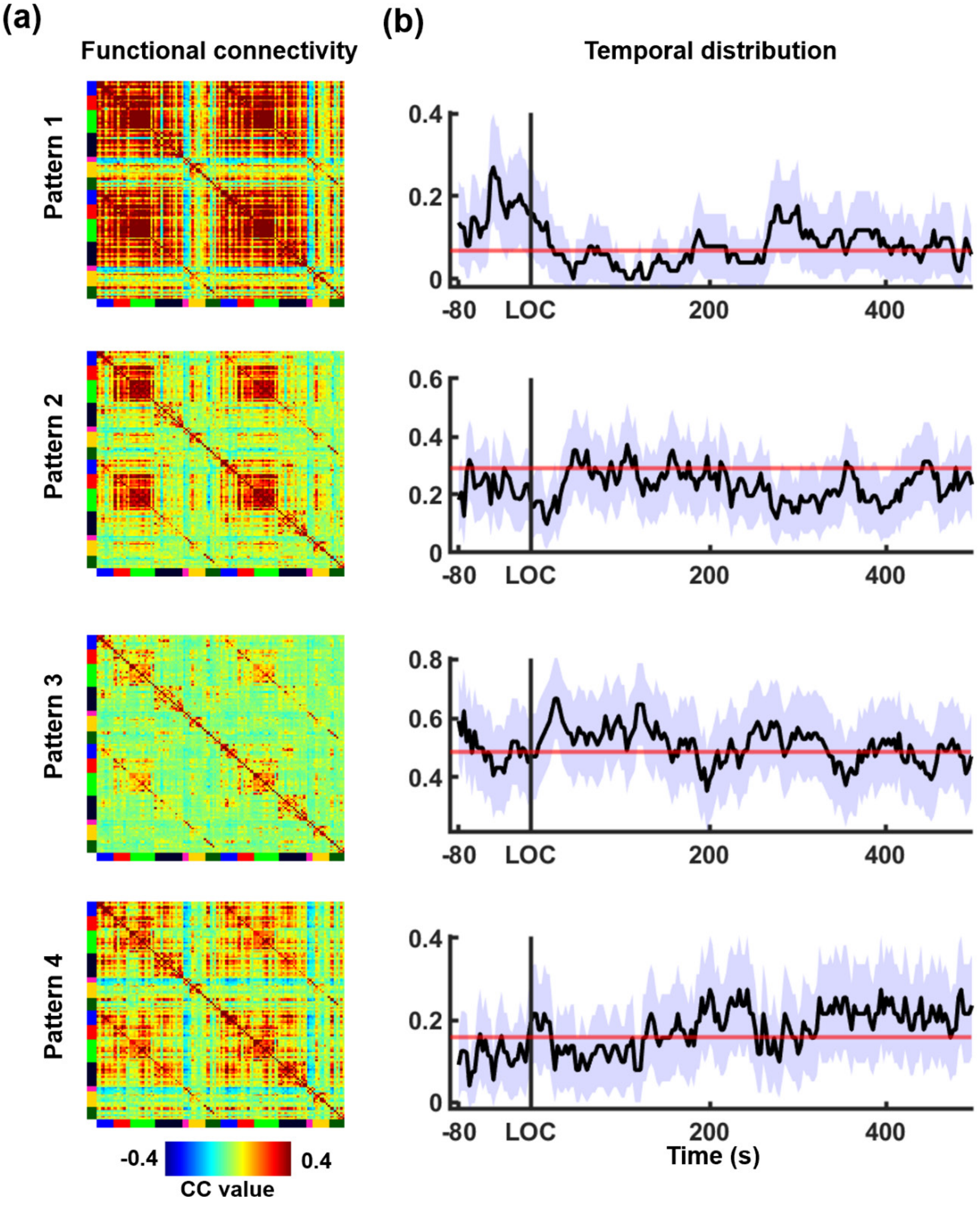

Figure S14. Dynamic brain states under different consciousness conditions with a cluster number of 4. (**a**) Four brain states, identified by unsupervised clustering of dynamic functional connectivity matrices. (**b**) Temporal distributions of four brain states around LOC and under the sustained unconsciousness. The purple shade indicates 95% confidence interval. The red line represents the averaged temporal distribution under low-dose propofol (i.e. baseline).

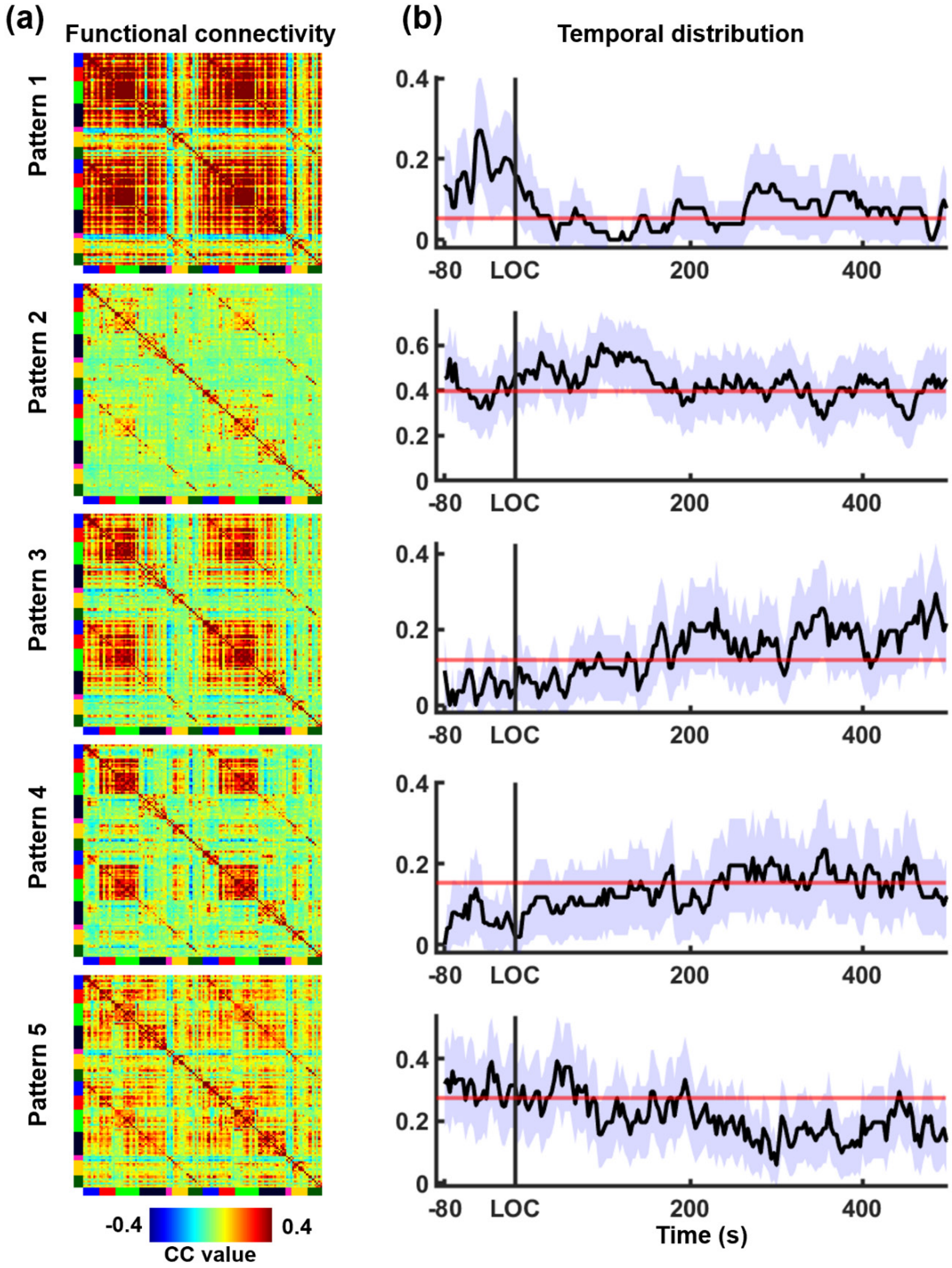

Figure S15. Dynamic brain states under different consciousness conditions with cluster number of 5. (**a**) Five brain states, identified by unsupervised clustering of dynamic functional connectivity matrices. (**b**) Temporal distributions of five brain states around LOC and under the sustained unconsciousness. The purple shade indicates 95% confidence interval. The red line represents the averaged temporal distribution under low-dose propofol (i.e. baseline).

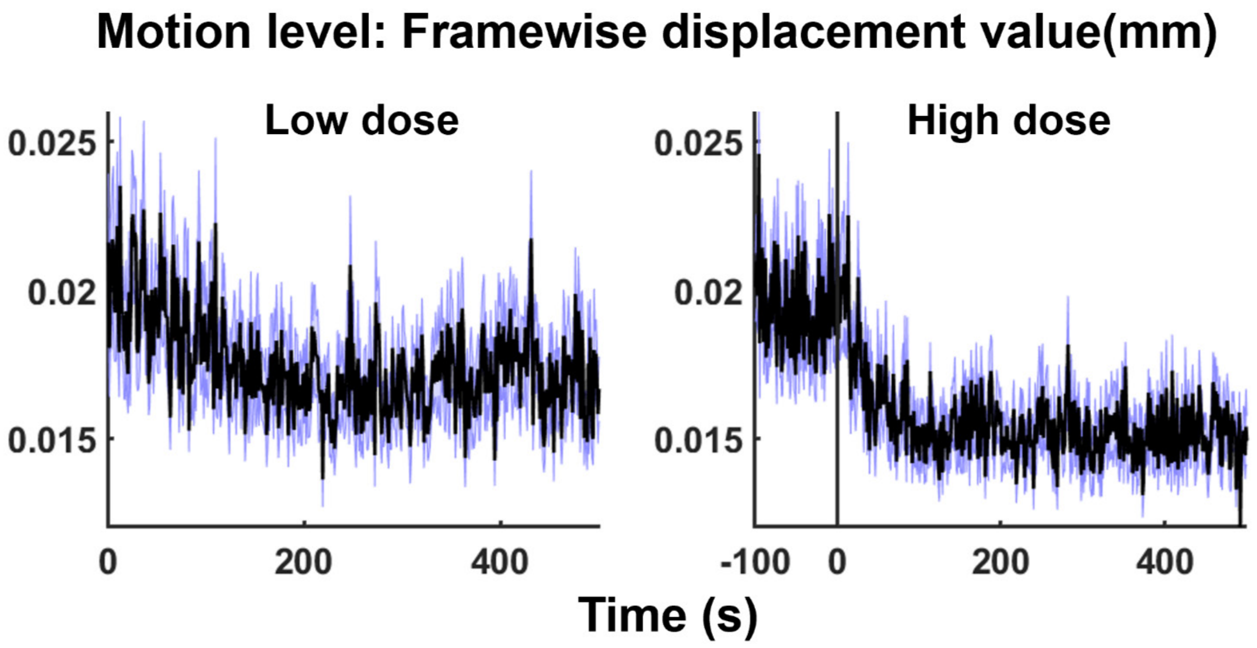

Figure S16. Motion level under (a) low-dose propofol and (b) around loss of consciousness. The moment of LOC is defined as time 0 for the second scan (i.e. high dose).

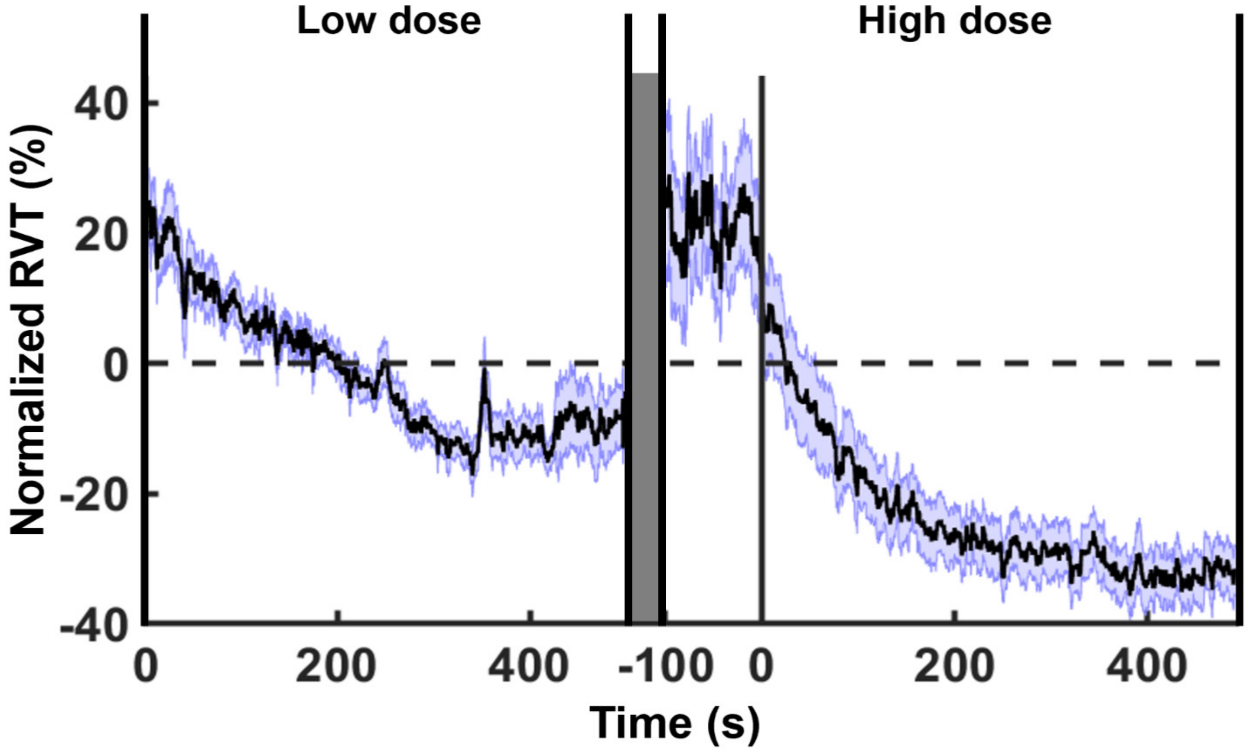

Figure S17. Respiration during graded propofol. The moment of LOC is defined as time 0 for the second scan (i.e. high dose).
